# Can Natural Products be Used for Protection of the Liver?

**Published:** 2013-05-04

**Authors:** Mohammad Hassan Pipelzadeh

**Affiliations:** 1 Department of Pharmacology and Toxicology Research Centre, Ahvaz Jundishapur University of Medical Sciences, Ahvaz, IR Iran

**Keywords:** Liver, Biological Agents, Safety, Toxicity

Industrial and technological advancements, particularly in the last century have produced astronomical changes in the environmental conditions that directly affect our health. The liver is the major site for detoxification of the harmful chemicals and some drugs that are taken regularly (e.g. acetaminophen) or in high doses. Prolonged exposure to such agents can result in development of various liver diseases worldwide. Many drugs commonly used for treatment of various diseases and metabolized or activated by the liver by first pass effects. These not only do not offer protection, but also are agents that impose a great burden of the liver and expose it to damaging effects by their toxic metabolites. Furthermore, despite the great advances in modern medicine, there is no effective drug available that stimulates liver function, protect it from damage or help to regenerate hepatic cells (1). There is, therefore, urgent need for effective drugs to replace those in current use or administered as supplements to protect the liver for the insults of these chemicals we use for treatment of an ailment we suffer from. As an alternative, the plant kingdom is undoubtedly valuable as a source of new medicinal agents. Historically may medicinal drugs have their origin from natural sources and plant products. Penicillin and many other newer antibacterial agents, currently in use in fighting bacterial infections, and the *vinca* alkaloids (vincristine and vinblastine), used in treatment of cancer, are good examples of such wealth that natural products can offer. It would be naive to assume that every product traditionally administered for many years before and has a natural origin is safe and effective in treatment of a given disease. Authorities in different countries, including WHO, and rightly so, require that safety issues to be address first in the preliminary approval of these products before their use is granted. 

However, 80% of the world population, due to economic restrains and unavailability of new medicinal products, rely on the use of traditional medicine, which is predominantly based on plant material (2). Therefore, it is the duty of researchers worldwide to provide scientific evidence of the safety and effectiveness before they are brought to wide scale use in treatment of any disease in human or animal. In line with this principle aim of this Journal (JJNPP), in this issue we published two papers that focus on and hepatoprotective effectiveness of *Echinacea purpurea* on liver function and the safety (mutagenic potential) of *Anethum graveolens*. In the first paper, Annahita Rezaie et al., report their research results on the protective effects of Echinacea purpurea on hepatic and renal toxicity induced by diethylnitrosamine in rats. Although the results are not conclusive in terms of the safety issues related to *Echinsea* when given alone (it reduced ALT, Creatinine, and direct bilirubin, and increased AST, ALP and Total bilirubin), it is shown to have a role in protection against the toxic manifestations of the diethylnitrosamine (a very toxic agent and is implicated in development of liver cancer) after a 30 day treatment in rat. In the second paper, *Kalantari* et al., the mutagenic potential of dill (Anethum graveolens), commercially available product in Iran under trade name *Dillsun*, was assessed under in vitro conditions. The method used for assessment of mutagenic potential was based gel electrophoresis. When compared with sodium dichromate, a potent and standard agent tested for mutagenesis experiments, *Dillsun* was found not to have such effect at low doses but at higher doses in a produced marginal increase in mutagenic index. Put together, although the findings of these research papers, publisher in this issue of the Journal, highlight the importance for further studies that need to be undertaken in order to assure safety and measures that need to be considered when these agents are administered at long term to human population.
